# Convergent Validity of Multiple 24‐h Dietary Recalls and Food Frequency Questionnaire in Calculating the Dietary Inflammatory Index Score in Community‐Dwelling Older Adults

**DOI:** 10.1002/fsn3.71501

**Published:** 2026-01-29

**Authors:** Corey Linton, Mia A. Schaumberg, Hattie H. Wright

**Affiliations:** ^1^ School of Allied Health Sciences, Faculty of Health Charles Darwin University Brinkin Northern Territory Australia; ^2^ School of Health University of the Sunshine Coast Sippy Downs Queensland Australia; ^3^ Sunshine Coast Health Institute Birtinya Queensland Australia; ^4^ Thompson Institute University of the Sunshine Coast Birtinya Queensland Australia; ^5^ Manna Institute University of the Sunshine Coast Sippy Downs Queensland Australia; ^6^ School of Human Movement and Nutrition Sciences The University of Queensland St Lucia Queensland Australia

**Keywords:** 24 h recall, dietary inflammatory index, dietary methods, DII, food frequency questionnaire, validity

## Abstract

Reliable dietary assessment methods underpin the confidence in reported dietary outcome measures including a priori dietary pattern indexes such as the dietary inflammatory index (DII) score. The food frequency questionnaire (FFQ) is commonly used to gather dietary data from which the DII is calculated. The FFQ, however, requires recall of dietary intake over several months. This study aimed to (i) determine the comparability of DII scores calculated from multiple 24 h recalls versus a FFQ, and (ii) identify the number of 24 h recalls required for a comparable DII score. Dietary data were collected from *n* = 94 community‐dwelling older adults in Australia (73.1 ± 4.8 years, 70.2% female) by an accredited practicing dietitian using four 24 h recalls over a two‐week period and a FFQ. Convergent validity was assessed between each possible comparative product of calculated DII scores by Pearson correlation, paired *t*‐test, absolute difference, and Bland Altman analysis. Most participants had a healthy body weight for their age (average BMI = 25.9 ± 4.0 kg/m^2^), were physically active (*n* = 91.5%), and were highly educated (Vocational education = 9.6%, Tertiary education = 64.9%). There were positive correlations between the FFQ and one (*r* = 0.219, *p* = 0.034), two (*r* = 0.205, *p* = 0.072), three (*r* = 0.334, *p* = 0.003), and four (*r* = 0.444, *p* ≤ 0.001) 24 h recalls. Bland–Altman plots demonstrated that four 24 h recalls exhibited the closest alignment with FFQ derived DII scores. As the number of recalls increased, the DII scores became more comparable to those from the FFQ. In community‐dwelling older adults, utilizing a minimum of four 24 h recalls to calculate DII scores are comparable to scores calculated from a FFQ.

## Graphical abstract

This study examined the validity of using multiple 24‐h dietary recalls to calculate the Dietary Inflammatory Index (DII) compared to a food frequency questionnaire (FFQ) in community‐dwelling older adults. Bland–Altman plots demonstrated that four 24 h recalls exhibited the closest alignment with FFQ derived DII scores. In community‐dwelling older adults, utilizing a minimum of four 24 h recalls to calculate DII scores is comparable to scores calculated from a FFQ.

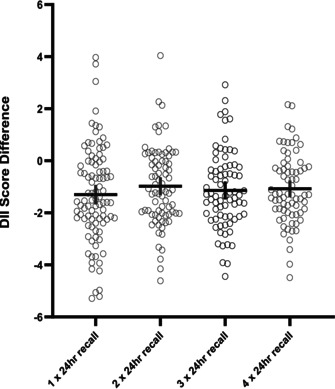

## 1 Introduction

A priori dietary patterns are one method by which the quality of a whole diet can be evaluated. This involves a structured technique by which a dietary index tool evaluates the diet of a specific population. The development of a priori dietary index tools is based on existing evidence (e.g., expert consensus and epidemiological data) to identify key nutrients, food, and/or food groups associated with the health condition the tool is developed to examine (Burggraf et al. 2018). Dietary indices or scoring systems are then used to examine the associations between the whole diet and a specific health outcome (Burggraf et al. 2018; Kourlaba and Panagiotakos 2009). The dietary inflammatory index (DII) is an example of a theory informed a priori dietary index tool designed to evaluate the inflammatory potential of a diet (Shivappa et al. 2014a). The overall score from the DII algorithm combines the effect of dietary parameters on inflammation and indicates an overall pro‐inflammatory dietary pattern with a positive score and an anti‐inflammatory dietary pattern with a negative score (Shivappa et al. 2014a). The use of the DII in research has increased substantially in recent years; however, there is currently no suggested method for dietary data collection to prospectively calculate the DII. Specifically in older adults, the DII shows promising potential for evaluating dietary patterns that support healthy aging by reducing chronic inflammation. Reliability of a priori dietary index tools, such as the DII, is in part dependent on the accuracy of the dietary data collected which in turn is dependent on using the most appropriate dietary assessment method to collect dietary data from the population being investigated. To date, most studies examining the inflammatory potential of the diet with the DII have collected dietary data through a food frequency questionnaire (FFQ) (Azarmanesh et al. 2023; Tabung et al. 2015; Vahid et al. 2018; Wirth et al. 2017). Although there are several studies which utilize different dietary data collection methods such as diet recalls and 24 h recalls, there is no consensus recommendation of method within the literature.

Assessment of dietary intake is a complex and intricate process that presents several challenges (Slimani et al. 2015). Nevertheless, the collection of reliable dietary information is key when examining the role of diet in health outcomes (Bingham 2002; Freedman et al. 2011). A FFQ and 24 h recalls are examples of retrospective dietary assessment methods which rely on memory recall and are based on self‐reported data (Bingham 1991). Limitations to both assessment methods include recall bias and social desirability bias which may result in under‐ or overreporting of foods and nutrients (Grandjean 2012; Larson 2019), ultimately influencing the validity of the dietary data (Adamson et al. 2009; Naska et al. 2017). An advantage of the FFQ is its ability to capture long‐term dietary patterns efficiently. This dietary assessment tool, however, has limited flexibility, as participants are required to select from a country‐specific list of predefined foods, beverages, and meals. In addition, it may lack detail on specific food preparation methods that influence nutrient composition (Briefel et al. 1992). Another advantage of a FFQ is the practicality for large population studies, as a FFQ requires less time and resources to administer and analyze compared to other dietary assessment methodologies. However, usability of the FFQ is limited by in‐country availability and is often not freely available. In large cross‐sectional and epidemiological studies, these FFQ per‐unit costs can accumulate rapidly, presenting a considerable financial burden for researchers and may restrict access to those with limited funding.

Conversely, a 24 h recall is an attractive alternative dietary assessment method as it allows for inclusion of any food items and recipes and only requires recall of dietary intake of the past 24 h. Collection of multiple 24 h recalls has shown to provide a valid representation of habitual dietary intake when the multiple pass method is used (Bingham 1991; Moshfegh et al. 2008). Although 24 h recalls are often considered a flexible, adaptable, and affordable method for dietary assessment, they are not without cost. Nevertheless, the 24 h recall method offers a universally accessible dietary assessment method for researchers to utilize and apply the DII dietary index tool. Additionally, 24 h recalls carry a lower risk of recall bias when using the multiple pass method, which is especially beneficial when studying the dietary intake of older adult populations who may face challenges with long‐term memory or navigate complex food questionnaires (Azarmanesh et al. 2023; Bingham 1991; Briefel et al. 1992; Moshfegh et al. 2008).

Therefore, the aim of the present study was to first determine the comparability of the DII score calculated from dietary data captured from multiple 24 h recalls compared to dietary data captured from a FFQ, and secondly to identify the number of 24 h recalls needed to yield similar DII scores to that of a FFQ.

## 2 Methods

### 2.1 Study Population

This study used data from a larger community evaluation study, of which the recruitment strategy and participant demographics are detailed elsewhere (Linton et al. 2024, 2022). Briefly, apparently healthy, independent community dwelling older adults free from listed medical conditions or chronic communicable infectious diseases ranging in age from 65 to 85 years were recruited between June 2023 and March 2024. Participants were recruited from community‐based exercise classes, email lists, web‐based advertising, presentations, and by word‐of‐mouth. All participants were classified as “not high risk of experiencing a cardiac event during exercise” according to the adult pre‐exercise screening system (Norton and Norton 2011), and those that did not possess the physical capability to engage in daily activities without significant limitations were excluded. Participants completed an online survey gathering socio‐demographic information including age, gender, marital status, and income, administered via Qualtrics. Leisure time physical activity frequency was assessed with the Godin Leisure‐Time questionnaire (Amireault and Godin 2015). This research received ethical approval from the University of the Sunshine Coast Human Research Ethics Committee under the reference number #A201498.

### 2.2 Assessment of Dietary Intake

Dietary data were collected by an accredited practicing dietitian using four 24 h recalls and a FFQ (Collins et al. 2014). The four dietary recalls per participant were collected using the multiple pass method. Briefly, this method requires the participant to provide the interviewer with a quick list of all foods consumed during the previous 24 h; this is followed by a second pass using a cross‐check of commonly forgotten foods. The third pass gains detail on the time and occasion of eating, and the fourth pass obtains a detailed description of each food and reviews eating occasions for omissions. Finally, a fifth pass is a readback of collected information which seeks to identify any remaining foods, including small or easily forgotten items (Moshfegh et al. 2008). The initial 24 h recall was collected in person and thereafter via the phone on nonconsecutive days within a two‐week period. Specifically, there was a minimum of 2 days between each 24‐h dietary recall, including at least one weekend day, and participants were unaware of the days these were to be conducted. Food portion sizes were assessed using standardized serving sizes and household measurement units as well as a food model booklet to facilitate accurate quantification of their food and beverage intake (Australian Bureau of Statistics 2015). Dietary data collected from the FFQ was completed online on the same day as the initial 24 h recall. The Australian Eating Survey was the FFQ used, which is a 120‐item semi‐quantitative FFQ (Collins et al. 2014). Standard portion sizes for adult men and women were established for each food item based on data from the most recent National Nutrition Survey (McLennan and Podger 1998), using unpublished data purchased from the Australian Bureau of Statistics, as well as “natural” serving sizes for common foods, such as a slice of bread (Collins et al. 2014). Respondents provided individual responses for each food or food type, with frequency options ranging from “Never” to “four or more times per day,” and for certain beverages, up to “seven or more glasses per day,” depending on the item (Collins et al. 2014). The FFQ categorized food items into specific groups, including beverages, breads and cereals, dairy products, main meals, sweets and snacks, and fruits and vegetables (Collins et al. 2014). Dietary data were manually entered into FoodWorks Professional Version 10, an Australian nutrient analysis software that utilizes the AUSNUT 2011–2013 food composition database (Neale et al. 2016). Following data entry, energy, nutrient, and food group intake were exported to Excel for subsequent analysis in SPSS.

### 2.3 Calculation of the Dietary Inflammatory Index (DII)

The DII was calculated five times for each participant (one 24 h recall, the average of two 24 h recalls, the average of three 24 h recalls, the average of four 24 h recalls and the FFQ). Dietary data exported from FoodWorks into Excel was imputed into the developed DII calculation tool; the methods for the development of the tool are described elsewhere (Shivappa et al. 2014a). Briefly, the DII assesses the inflammatory potential of the diet, identifying both pro‐ and anti‐inflammatory diets (Shivappa et al. 2014a). The tool utilizes the consumption of 45 foods, nutrients and nonnutrients alongside their accompanied global means and standard deviations (SD) to calculate a food specific inflammatory value (Shivappa et al. 2014a). The cumulative score from the DII captures the combined effect of dietary components on inflammation, where a more negative score indicates an anti‐inflammatory diet and a more positive score indicates a pro‐inflammatory dietary pattern with a potential score ranging from −8.87 to 7.98 (Hébert et al. 2019; Shivappa et al. 2014a). For the present study, garlic, ginger, saffron, turmeric, pepper, thyme/oregano, and rosemary were not included in calculating the DII score due to the difficulty in quantifying portion sizes. In addition, vitamin D, flavan‐3‐ol, flavones, flavanols, flavanones, anthocyanidins and isoflavones were not included due to consumption values not being calculated in FoodWorks. Others have also excluded similar foods and nonnutrients from calculated DII scores due to quantification difficulty (Bagheri et al. 2020; Fowler and Akinyemiju 2017; Linton et al. 2022; Orchard et al. 2017).

### 2.4 Statistical Analysis

Normality of data distribution was assessed via the Shapiro Wilk test. Data that were not normally distributed were transformed using the natural Log or Log base 10 and normality was assessed again. All continuous variables are expressed as means ± (SD) and categorical data are expressed as frequencies and percentages; DII is expressed as a continuous variable. Comparability of DII scores from multiple 24 h recalls to the FFQ is assessed through reporting the mean absolute and percentage difference, confidence intervals (95%) between the DII score calculated from the FFQ, four 24 h recalls and subsequent 24 h recalls. To accurately determine convergent validity and comparability of DII scores calculated from multiple 24 h recalls to the FFQ, multiple statistical tests were compared (Lombard et al. 2015). As outlined by Lombard et al. (2015), no single statistical test sufficiently captures all facets of validity; therefore, convergent validity was assessed between each possible comparative product of calculated DII scores by Pearson correlation, paired *t*‐test, absolute difference, and Bland Altman analysis.

The DII was initially validated using three 24 h recalls and again in postmenopausal women using a FFQ, therefore for the present study DII scores calculated from both the FFQ and four 24 h recalls were used as the reference calculations (Shivappa et al. 2014b; Tabung et al. 2015). Pearson correlation statistics assessed the strength and direction of associations between the DII score from the reference calculations (FFQ and four 24 h recalls) and all comparators (24 h recall 1, average of 24 h recall 1 and 2, average of 24 h recall 1, 2, and 3). As described in the literature, others have deemed an acceptable outcome to be *r* ≥ 0.20 (Lombard et al. 2015). An acceptable *r* ≥ 0.20 recognizes the inherent variability and complexity in self‐reported dietary data whilst allowing for the minute variable change in absolute DII score. Paired *t*‐test assessed the agreement between the DII score from the reference value to the three comparators. An acceptable outcome was determined to be *p* > 0.05 (Lombard et al. 2015). A nonsignificant result (*p* > 0.05) from a *t*‐test indicates only a failure to detect a difference between means, but it does not confirm that the two measures are equivalent. In contrast, true equivalence testing reverses the null hypothesis, assuming the two values are different and only concludes equivalence when there is strong evidence that the difference lies within an a priori‐defined equivalence margin. The Bonferroni correction was applied to adjust for multiple comparisons, reducing the risk of Type I errors by adjusting the *p*‐values for each statistical test according to the number of comparisons made (Weisstein 2004). Absolute difference assessed the difference in DII score between the reference calculations and the three comparators. Bland Altman analysis explored the absolute agreement of methods to the comparator calculations with an acceptable error of 5% DII (Bland and Altman 1986). Bland Altman analysis evaluates the practical difference in DII scores to predict a meaningful change in inflammatory biomarkers (Shivappa et al. 2014a, 2014b). This multi‐method approach provides a robust framework for assessing convergent validity, as recommended by Lombard et al. (2015), and accounts for both statistical and practical relevance in dietary assessment. All data were analyzed using Microsoft Excel 2019 and SPSS (version 22.0, SPSS Inc., Chicago, IL, USA) with significance set at *p* < 0.05 unless otherwise stated.

## 3 Results

### 3.1 Participant Characteristics

A sample of *n* = 94 older adults (73.1 ± 4.8 years, 70.2% female) was recruited; participant characteristics are presented in Table 1. Most completed tertiary education, were sufficiently active, and had a healthy BMI for their age (Javed et al. 2022). There were no significant differences for age and activity levels between males and females; however, males had a larger BMI than females (27.3 ± 3.7 kg/m^2^ vs. 25.2 ± 4.0 kg/m^2^, *p* = 0.021).

**TABLE 1:** Participant characteristics of total cohort and grouped by sex.

	Total (*n* = 94)	Males	Females	*p*
Age (years)	73.1 ± 4.8	74.0 ± 4.8	72.7 ± 4.8	0.219
Sex (% female)	66 (70.2)	—	—	—
BMI (kg/m^2^)	25.9 ± 4.0	27.3 ± 3.7	25.2 ± 4.0	0.021
Leisure time exercise, *n* (%)				
Insufficiently/moderately active^a^	8 (8.5)	1 (1.1)	7 (7.4)	0.428
Active^b^	86 (91.5)	27 (28.7)	59 (62.8)	
Highest level of education, *n* (%)				
Primary/secondary education	24 (25.5)	6 (6.4)	18 (19.1)	0.831
Vocational education	9 (9.6)	3 (3.2)	6 (6.4)	
Tertiary education	61 (64.9)	19 (20.2)	42 (44.7)	

Abbreviation: BMI, body mass index.

^a^ Godin score ≤ 23 (Amireault and Godin 2015).

^b^ Godin score ≥ 24 (Amireault and Godin 2015).

### 3.2 Comparability of DII Calculations

The comparability and convergent validity of DII scores calculated from various 24 h recalls compared to the DII scores calculated from the FFQ are presented in Table 2. All participants completed one 24 h recall whilst less completed two (*n* = 78, 83.0%), three (*n* = 75, 80.0%), and four (*n* = 70, 74.5%) recalls. The DII scores calculated from the FFQ, three, and four 24 h recalls are positively correlated and one and two recalls are not. However, the average DII scores calculated from 24 h recalls were significantly different compared to the FFQ (FFQ mean DII = −1.42). The percent and absolute difference between the DII calculated from the FFQ and all 24 h recalls fell outside of the acceptable limits (Lombard et al. 2015).

**TABLE 2:** Agreement of DII scores calculated from 24 h recalls compared with DII scores calculated from the FFQ.

	Mean DII	Pearsons correlation		Paired *t*‐test	Mean difference		
		*r*	*p* ^a^	*p* ^a^	% of raw	Absolute	95% CI
1 × 24 h recall (*n* = 94)	−0.13 ± 1.7	0.219	0.068	< 0.001	−111.63	−1.30	−1.67, −0.92
2 × 24 h recall (*n* = 78)	−0.48 ± 1.3	0.205	0.144	< 0.001	−101.26	−0.98	−1.32, −0.64
3 × 24 h recall (*n* = 75)	−0.30 ± 1.4	0.334	0.006	< 0.001	−131.29	−1.14	−1.48, −0.80
4 × 24 h recall (*n* = 70)	−0.39 ± 1.4	0.444	< 0.001	< 0.001	−103.75	−1.07	−1.42, −0.79

^a^ Bonferroni adjusted *p*‐value.

**FIGURE 1:**
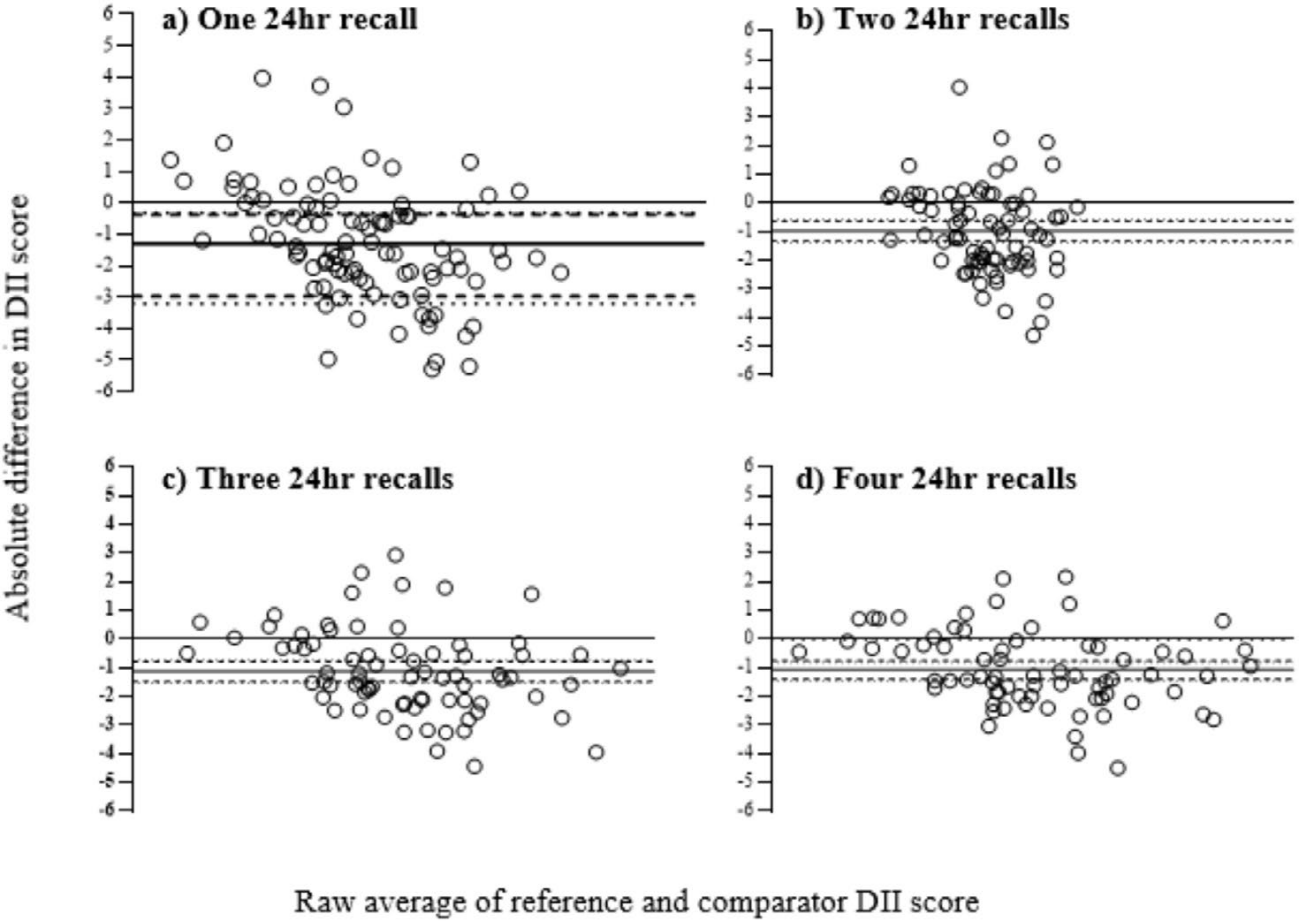
Bland and Altman plots displaying measurement agreement of multiple 24 h recalls compared to the DII calculated by the FFQ. Bland and Altman plots displaying measurement agreement to the DII calculated by the FFQ, expressed as absolute of the reference value (relative error) against the average of the DII from the FFQ and multiple 24 h recalls. (a) 1 × 24 h recall, (b) 2 × 24 h recalls, (c) 3 × 24 h recalls, (d) 4 × 24 h recalls. In this plot, the *x*‐axis represents the mean of the two measurement methods, while the *y*‐axis displays the difference between the two methods. Bias was calculated as average error and 95% limits of agreement as 2 SD from the line of bias, with an acceptable error of 5% DII, established a priori. Black line: Bias, dashed line: 95% limits of agreement, dotted line: Acceptable error.

The measurement of agreement of DII scores calculated from multiple 24 h recalls and the FFQ are displayed in Figure 1. The DII scores calculated had the potential to range from −8.87 to +7.98, where more negative values indicate an anti‐inflammatory diet and positive values represent a pro‐inflammatory diet (Hébert et al. 2019; Shivappa et al. 2014a). As more 24 h recalls are included in the calculation of the DII, scores become closer to the scores calculated from the FFQ. The repeatability index (a. 2.16, b. 0.358, c. 0.396, d. 0.350) indicates that as 24 h recalls are added the DII scores become progressively more comparable to the DII scores from the FFQ. The DII calculated from multiple 24 h recalls tends to underestimate the DII compared to the FFQ; however, as more 24 h recalls are added, the distribution of difference approaches closer to 0. Similarly, as more recalls are added, the 95% confidence intervals become smaller, indicating closer agreement to the DII calculated from the FFQ.

**FIGURE 2:**
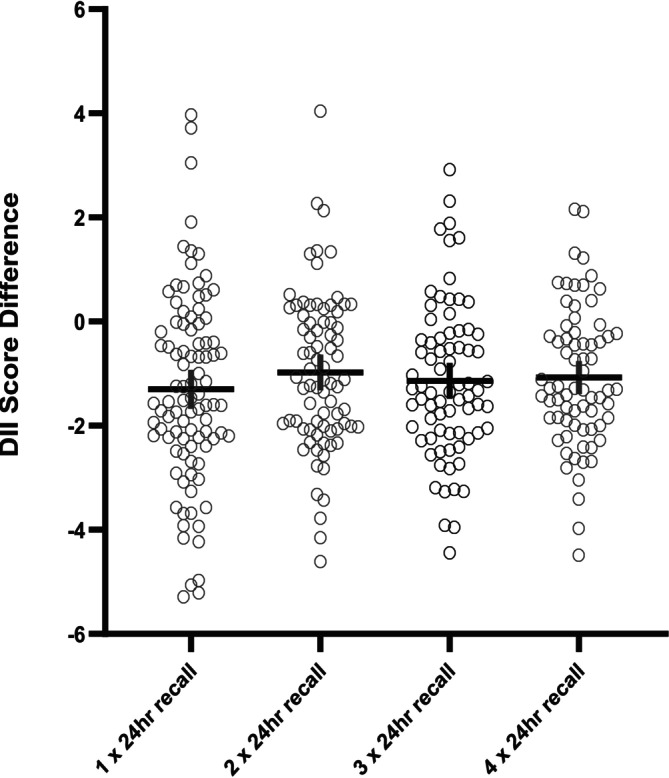
Mean comparison of DII scores calculated from multiple 24 h recalls to the DII scores calculated from the FFQ.

The mean differences in DII scores between multiple 24 h recalls and the reference calculation derived from the FFQ are presented in Figure 2. The mean differences are displayed to illustrate the variability and potential discrepancies in DII scores when using different dietary intake methodologies.

The comparability and convergent validity of DII scores calculated from one, two, or three 24 h recalls compared to the DII scores calculated from four 24 h recalls are presented in Table 3. All DII scores were significantly correlated, and the strongest correlation was found between the DII of three and four 24 h recalls.

**TABLE 3:** Agreement of DII scores, compared with DII scores calculated from four 24 h recalls.

	Pearsons correlation		Paired *t*‐test	Mean difference		
	*r*	*p* ^a^	*p* ^a^	% of raw	Absolute	95% CI
1 × 24 h recall (*n* = 71)	0.756	< 0.001	0.999	44.70	−0.04	−0.30, 0.22
2 × 24 h recall (*n* = 71)	0.760	< 0.001	0.544	−55.39	0.12	−0.10, 0.34
3 × 24 h recall (*n* = 71)	0.927	< 0.001	0.880	−17.38	−0.05	−0.18, 0.08

^a^ Bonferroni adjusted *p*‐value.

**FIGURE 3:**
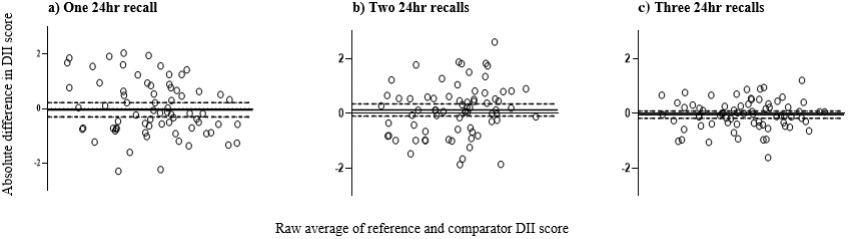
Bland and Altman plots displaying measurement agreement to the DII score calculated by four 24 h recalls. Bland and Altman plots displaying measurement agreement to the DII score calculated by four 24 h recalls, expressed as an absolute of the reference value (relative error) against the average of the DII score calculated from multiple 24 h recalls (a) 1 × 24 h recall, (b) 2 × 24 h recalls, (c) 3 × 24 h recalls. Bias was calculated as average error and 95% limits of agreement as 2 SD from the line of bias, with an acceptable error of 5% DII, established a priori. Black line: Bias, dashed line: 95% limits of agreement, dotted line: Acceptable error.

The measurement of agreement off DII scores calculated from one, two and three 24 h recalls and four 24 h recalls are displayed in Figure 3. As more 24 h recalls are included in the calculation of the DII, scores become closer to the scores calculated from the four 24 h recalls. The repeatability index (a. 1, b. 0.506, c. 0.377) indicates that as 24 h recalls are added the DII scores become progressively more comparable to the DII scores from four 24 h recalls. The DII calculated from multiple 24 h recalls tends to underestimate the DII compared to four 24 h recalls however, as more 24 h recalls are added the distribution of difference approaches closer to 0. Similarly, as more recalls are added, the 95% confidence intervals become smaller, indicating closer agreement to the DII calculated from four 24 h recalls.

**FIGURE 4:**
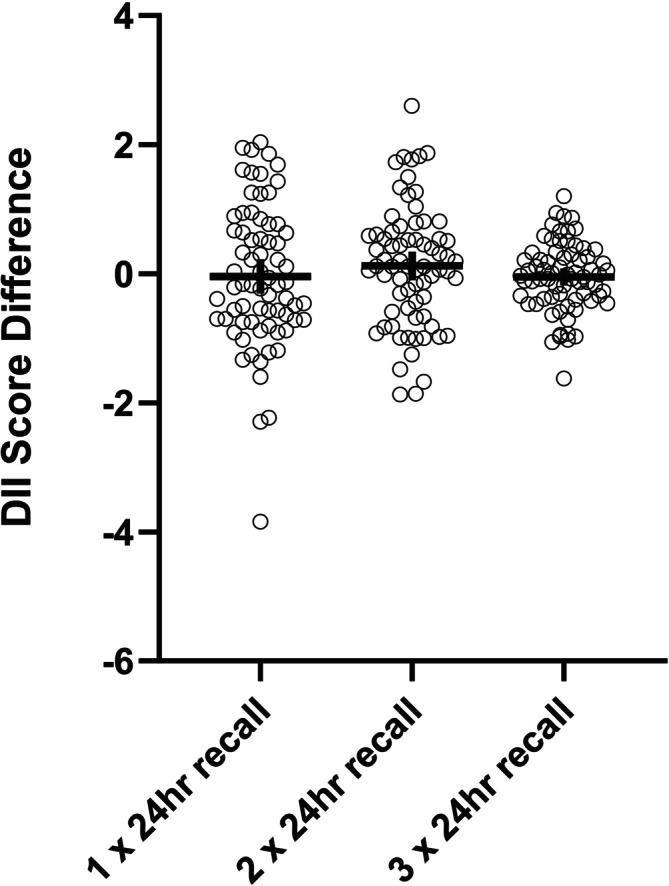
Mean comparison of DII scores calculated from multiple 24 h recalls to the DII scores calculated from the four 24 h recalls.

The mean differences in DII scores between one, two and three 24 h recalls and the reference calculation derived from four 24 h recalls are presented in Figure 4. The mean differences are displayed to illustrate the variability and potential discrepancies in DII scores when using different dietary intake methodologies.

## 4 Discussion

The findings of this study demonstrate that multiple 24 h recalls can provide a comparable estimation of the DII score to that derived from a FFQ, with stronger agreement observed as the number of 24 h recalls increased. Pearson correlation analyses showed that DII scores from three (*r* = 0.205, *p* = 0.006) and four (*r* = 0.334, *p* ≤ 0.001) 24 h recalls exhibited the strongest correlations with the FFQ derived DII scores. However, paired *t*‐tests indicated that DII scores from 24 h recalls were not statistically equivalent to FFQ derived DII scores (*p* < 0.001), suggesting some variability between methods. Bland–Altman analysis demonstrated that measurement agreement improved with additional recalls, with four 24 h recalls providing the closest approximation to the FFQ. Similarly, mean comparison confirmed that four 24 h recalls produced the most comparable DII scores to those from the FFQ. The second aim of this study sought to identify the number of 24 h recalls required to yield a comparable DII score to that of a FFQ. In the present findings, using three 24 h recalls yielded DII scores that were closest to those from four recalls. Analyses indicated that as 24 h recalls were added, DII scores became progressively closer to the DII calculated from four 24 h recalls. Similarly, the level of agreement also increased between different quantities of 24 h recalls and DII scores derived from four 24 h recalls as additional days recalls were added. This indicates that with each additional day of recall, the DII scores more closely approximated those derived from four recalls.

First, the comparability of the DII score calculated from multiple 24 h recalls compared to a FFQ using three methods to evaluate convergent validity was examined. Pearson correlation statistics were conducted, which found a positive correlation between DII scores derived from three (*r* = 0.334, *p* = 0.006) and four (*r* = 0.444, *p* ≤ 0.001) 24 h recalls and those calculated from the FFQ, with the strength of the correlation increasing as the number of recalls increased. Findings indicate that DII scores derived from four 24‐h recalls were moderately associated with those calculated from the FFQ (*r* = 0.444, *p* ≤ 0.001), suggesting that the two methods tend to rank individuals in a similar direction with respect to dietary inflammatory potential (Lombard et al. 2015). However, paired *t*‐test indicated that the DII scores calculated from 24 h recalls were not acceptable when compared to the FFQ (*p* ≤ 0.001) (Lombard et al. 2015). This may be because the size of the DII scores is small, which sees greater variability. For instance, a DII score of 0.2 may seem minor, but when compared to a score of 0.8 calculated from a different method, the relative difference is substantial, representing a fourfold variation in inflammatory potential. This highlights the sensitivity of the DII score, where even small numerical differences can translate into statistically meaningful variations in dietary inflammatory potential but perhaps not clinically meaningful differences. Previous research has consistently shown that higher DII scores calculated from both 24 h recalls and FFQs are associated with elevated inflammatory biomarkers such as CRP, IL‐6, and TNF‐α; however, the magnitude of these associations is typically modest (Hua et al. 2024; Lécuyer et al. 2023). Importantly, a pronounced change in DII score does not necessarily equate to a similarly substantial change in systemic inflammatory markers, as dietary inflammatory scores generally explain only a small proportion of the variance in biomarker levels.

The Bland–Altman analysis displayed the measurement agreement between the DII scores calculated by the FFQ and those from multiple 24 h recalls. Despite most data falling outside of the 95% CI and measures of error, four 24 h recalls were the closest to the DII scores calculated from the FFQ. This indicated that as more recalls were added the measure of agreement increased. These findings were reinforced by mean comparison, demonstrating that DII scores from four 24 h recalls were more comparable to the FFQ compared to fewer 24 h recalls. It is important to acknowledge that the potential impact of using more than four recalls was not assessed, and therefore, it remains uncertain whether a greater number of recalls would yield a DII score more closely aligned with that derived from the FFQ. Nevertheless, the findings underscore the importance of using multiple 24 h recalls, specifically four or more, to calculate DII scores that are comparable to those calculated from FFQs. This has practical implications for the interpretation of existing literature examining the association between dietary inflammation calculated with the DII and a health outcome. Specifically, studies that have used fewer than four 24 h recalls to calculate DII scores may yield estimates that are not directly comparable to DII scores calculated from an FFQ or studies using four or more 24 h recalls, thereby limiting comparison across studies (Ho et al. 2023; Li and Yuhong 2024; Linton et al. 2024; Linton et al. 2022; Mao et al. 2024; Zhai et al. 2024; Zhao et al. 2025).

Although the data show that additional recalls enhanced the comparability and reliability of the DII calculations, it must be noted that the burden on participants increases when more 24 h recalls are added for dietary data collection. Similar to others, this study found attrition in the amount of participants completing additional 24 h recalls likely due to the demands of completing multiple 24 h recalls which can lead to fatigue, decreased motivation, and potential attrition among participants (Hedrick et al. 2012). As the number of recalls increases, the likelihood of misreporting due to participant disengagement also increases, ultimately compromising the integrity of the data collected (Hedrick et al. 2012). Thus, researchers face the challenge of balancing the desire for true representation of usual dietary intake with the need to minimize participant burden. The findings of this study offer valuable insights into the use of multiple 24 h recalls for calculating DII scores, particularly in an older population with a higher level of education and in good health. Furthermore, 24 h recalls may reduce the risk of recall bias as they rely on short‐term memory and are typically interviewer‐led, allowing for real‐time clarification and prompting to enhance accuracy of dietary reporting. From a practical perspective, these findings suggest that multiple 24 h recalls could offer a feasible and cost‐effective alternative to FFQs for calculating the DII, particularly in settings where rapid data collection or lower participant burden is required. The use of recalls may also support clinical applications by enabling more flexible and responsive assessment of recent dietary intake, allowing dietitians or researchers to monitor dietary changes and provide timely feedback. While FFQs remain valuable for capturing habitual intake, multiple recalls may offer a quicker, adaptable, and potentially more cost‐effective option in specific contexts where FFQs are impractical or not suitable for participants.

### 4.1 Strengths and Limitations

In this study, we examined the convergent validity of multiple 24 h recalls to a FFQ for calculating the DII score. It is acknowledged that the FFQ is a subjective measure and indirect method to examine the validity of multiple 24 h recalls to evaluating a diet's inflammatory potential by means of the DII tool. Future validation studies could include inflammatory biomarkers such as CRP, IL‐6, and TNF‐α to objectively validate the accuracy of dietary intake reported through multiple 24 h recalls. Incorporating these biomarkers in future research will enhance the validation of multiple 24 h recalls as a reliable dietary assessment tool for calculating the DII score. Findings of this study may not be generalizable to other older populations, particularly those with lower literacy levels. The participants were considered homogenous in relation to health status as they did not present with major chronic conditions or significant variations in disease burden that could influence dietary intake and inflammatory status. Most participants were generally healthy or had only minor health conditions, reducing the likelihood of confounding effects from severe illness or metabolic disorders. It is worth noting that this population resides within a geographical location which boasts a large variety of food availability and good socioeconomic conditions, which significantly impacts the dietary patterns of its residents leading to a greater variety of foods consumed and a larger variance in dietary intake among participants. This diversity can influence the accuracy of dietary data collection (Freedman et al. 2014), understanding the local food environment is essential for accurately assessing dietary intake and its implications for health. The characteristics of the present sample also included a high proportion of participants with a tertiary education, likely contributed to the accuracy and reliability of the dietary assessments conducted. In populations with lower literacy, challenges such as misinterpretation of questions, difficulty in reporting dietary intake, and potential recall errors may arise, potentially leading to different outcomes. Studies have shown that dietary data from low‐literacy groups often underestimate energy and key nutrient intakes compared to more literate populations, influencing diet–disease associations (Grandjean 2012; Santos‐Báez et al. 2025; Subar et al. 2003). This, in turn, may alter calculated DII scores, potentially weakening observed associations with health outcomes. Nevertheless, these results are promising and suggest that multiple 24 h recalls can serve as a comparable alternative to FFQs in similar populations, provided the potential limitations are acknowledged. Although up to four 24 h recalls were included, the potential impact of collecting additional recalls was not assessed in the present study. It is therefore uncertain whether a greater number of recalls would have produced DII scores more closely aligned with those derived from the FFQ. Furthermore, a strength of this study is that 24 h recalls were interviewer led following the multiple pass method (Moshfegh et al. 2008), which limits the risk of reporting and recall errors of the dietary data (Poslusna et al. 2009).

The inclusion of 31 out of 45 potential dietary parameters in the calculation of the DII score may be viewed as a limitation of the present study. However, all DII scores were calculated using the same food parameters, making scores directly comparable. This does not allow for the exploration of how each method may differ in quantifying the excluded food parameters, which could have provided further insight into the accuracy of the methods. The majority of studies exploring the associations between DII and health outcomes also exclude the same parameters (garlic, ginger, saffron, turmeric, pepper, thyme/oregano, and rosemary) due to quantification difficulty. Vitamin D, flavan‐3‐ol, flavones, flavanols, flavanones, anthocyanidins, and isoflavones were not calculated due to food analysis software package constraints (Fowler and Akinyemiju 2017; Mazidi et al. 2017; Shivappa et al. 2016; Xu 2025). Further, the DII tool has its own limitations when determining the overall inflammatory effect of the diet of an individual, such as not accounting for supplement use or data skewing through the consumption of nutrient/energy dense foods (Hébert et al. 2019). These limitations are inherent to the tool itself; it is critical to acknowledge them as limitations in the present study. Future studies may improve upon this by incorporating dietary assessment and analysis methods to capture a greater number of DII parameters. This would allow for a more complete evaluation of the differences between methods in quantifying excluded food parameters.

Finally, the small sample size of the present study may have contributed to a type II error, where associations could have been missed due to insufficient statistical power. However, a priori sample size calculations were not performed for this study, due to the absence of comparable studies in this population and context; there were insufficient data to inform the assumptions required for an accurate power calculation, such as expected correlation coefficients or levels of agreement. Additionally, the limited sample size reduces the generalizability of the findings, as the results may not be representative of the broader population.

## 5 Conclusion

In conclusion, a DII score calculated from four or more 24 h recalls yields comparable DII scores to that of a semi‐quantified FFQ. In addition, the findings support the use of multiple 24 h recalls as an acceptable dietary assessment method from which the a priori DII score can be calculated in apparently healthy, community dwelling older adults. Dietary assessment methodology should be considered when interpreting studies examining dietary inflammation using the DII score as the method of dietary assessment may significantly impact the calculated DII score. Future research may further examine the validity of multiple 24 h recalls as a dietary assessment method to accurately calculate a DII score and examine the inflammatory potential of a diet among older adults by including inflammatory biomarkers as an objective measure of inflammation.

## Author Contributions

**Corey Linton:** conceptualization (equal), data curation (lead), formal analysis (lead), investigation (lead), methodology (equal), project administration (equal), writing – original draft (lead), writing – review and editing (equal). **Mia A. Schaumberg:** conceptualization (equal), funding acquisition (lead), methodology (supporting), project administration (supporting), supervision (equal), writing – review and editing (equal). **Hattie H. Wright:** conceptualization (equal), formal analysis (supporting), methodology (supporting), supervision (equal), writing – review and editing (equal).

## Funding

This research was funded by a University of the Sunshine Coast—Sunshine Coast Council Regional Partnership Agreement (USC‐SCC RPA) Project Grant. C.L. is supported by the Australian Government Research Training Program Scholarship. The funders had no role in the design of the study; in the collection, analyses, or interpretation of data; in the writing of the manuscript, or in the decision to publish the results.

## Disclosure

Declaration of Generative AI and AI‐assisted technologies in the writing process: During the preparation of this work, the authors have not used generative AI or AI‐assisted technologies in the writing process.

## Conflicts of Interest

The authors declare no conflicts of interest.

## Data Availability

Data described in the manuscript, code book, and analytic code will be made available upon request pending accordance with the ethical principles.

## References

[fsn371501-bib-0001] Adamson, A. , J. Collerton , K. Davies , et al. 2009. “Nutrition in Advanced Age: Dietary Assessment in the Newcastle 85+ Study.” European Journal of Clinical Nutrition 63, no. 1: S6–S18. 19190647 10.1038/ejcn.2008.60

[fsn371501-bib-0002] Amireault, S. , and G. Godin . 2015. “The Godin‐Shephard Leisure‐Time Physical Activity Questionnaire: Validity Evidence Supporting Its Use for Classifying Healthy Adults Into Active and Insufficiently Active Categories.” Perceptual and Motor Skills 120, no. 2: 604–622. 25799030 10.2466/03.27.PMS.120v19x7

[fsn371501-bib-0003] Australian Bureau of Statistics . 2015. “Australian Health Survey: Users' Guide, 2011–13.” https://www.abs.gov.au/AUSSTATS/abs@.nsf/DetailsPage/4363.0.55.0012011‐13.

[fsn371501-bib-0004] Azarmanesh, D. , J. Pearlman , E. T. Carbone , J. C. DiNatale , and E. R. Bertone‐Johnson . 2023. “Construct Validation of the Dietary Inflammatory Index (DII) Among Young College‐Aged Women.” Nutrients 15, no. 21: 4553. 37960206 10.3390/nu15214553 PMC10647813

[fsn371501-bib-0005] Bagheri, A. , S. Soltani , R. Hashemi , R. Heshmat , A. D. Motlagh , and A. Esmaillzadeh . 2020. “Inflammatory Potential of the Diet and Risk of Sarcopenia and Its Components.” Nutrition Journal 19: 1–8. 33248463 10.1186/s12937-020-00649-2 PMC7700703

[fsn371501-bib-0007] Bingham, S. A. 1991. “Limitations of the Various Methods for Collecting Dietary Intake Data.” Annals of Nutrition and Metabolism 35, no. 3: 117–127. 1952811 10.1159/000177635

[fsn371501-bib-0006] Bingham, S. A. 2002. “Biomarkers in Nutritional Epidemiology.” Public Health Nutrition 5, no. 6a: 821–827. 12638591 10.1079/phn2002368

[fsn371501-bib-0008] Bland, J. M. , and D. Altman . 1986. “Statistical Methods for Assessing Agreement Between Two Methods of Clinical Measurement.” Lancet 327, no. 8476: 307–310. 2868172

[fsn371501-bib-0009] Briefel, R. R. , K. M. Flegal , D. M. Winn , C. M. Loria , C. L. Johnson , and C. T. Sempos . 1992. “Assessing the Nation's Diet: Limitations of the Food Frequency Questionnaire.” Journal of the American Dietetic Association 92, no. 8: 959–963. 1640039

[fsn371501-bib-0010] Burggraf, C. , R. Teuber , S. Brosig , and T. Meier . 2018. “Review of a Priori Dietary Quality Indices in Relation to Their Construction Criteria.” Nutrition Reviews 76, no. 10: 747–764. 30053192 10.1093/nutrit/nuy027 PMC6130981

[fsn371501-bib-0011] Collins, C. E. , M. M. Boggess , J. F. Watson , et al. 2014. “Reproducibility and Comparative Validity of a Food Frequency Questionnaire for Australian Adults.” Clinical Nutrition 33, no. 5: 906–914. 24144913 10.1016/j.clnu.2013.09.015

[fsn371501-bib-0012] Fowler, M. E. , and T. F. Akinyemiju . 2017. “Meta‐Analysis of the Association Between Dietary Inflammatory Index (DII) and Cancer Outcomes.” International Journal of Cancer 141, no. 11: 2215–2227. 28795402 10.1002/ijc.30922 PMC6056732

[fsn371501-bib-0013] Freedman, L. S. , J. M. Commins , J. E. Moler , et al. 2014. “Pooled Results From 5 Validation Studies of Dietary Self‐Report Instruments Using Recovery Biomarkers for Energy and Protein Intake.” American Journal of Epidemiology 180, no. 2: 172–188. 24918187 10.1093/aje/kwu116 PMC4082341

[fsn371501-bib-0014] Freedman, L. S. , A. Schatzkin , D. Midthune , and V. Kipnis . 2011. “Dealing With Dietary Measurement Error in Nutritional Cohort Studies.” Journal of the National Cancer Institute 103, no. 14: 1086–1092. 21653922 10.1093/jnci/djr189 PMC3143422

[fsn371501-bib-0015] Grandjean, A. C. 2012. “Dietary Intake Data Collection: Challenges and Limitations.” Nutrition Reviews 70, no. S2: S101–S104. 23121343 10.1111/j.1753-4887.2012.00545.x

[fsn371501-bib-0016] Hébert, J. R. , N. Shivappa , M. D. Wirth , J. R. Hussey , and T. G. Hurley . 2019. “Perspective: The Dietary Inflammatory Index (DII)—Lessons Learned, Improvements Made, and Future Directions.” Advances in Nutrition 10, no. 2: 185–195. 30615051 10.1093/advances/nmy071 PMC6416047

[fsn371501-bib-0017] Hedrick, V. E. , A. M. Dietrich , P. A. Estabrooks , J. Savla , E. Serrano , and B. M. Davy . 2012. “Dietary Biomarkers: Advances, Limitations and Future Directions.” Nutrition Journal 11: 1–14. 23237668 10.1186/1475-2891-11-109 PMC3568000

[fsn371501-bib-0018] Ho, F. K. , M. D. Wirth , S. Parra‐Soto , et al. 2023. “Dose‐Response Associations of Dietary Inflammatory Potential With Health Outcomes: A Prospective Cohort Study of 198,265 UK Biobank Participants.” 48, no. 9: 101774. 10.1016/j.cpcardiol.2023.101774 37121456

[fsn371501-bib-0019] Hua, R. , G. Liang , and F. J. M. Yang . 2024. “Meta‐Analysis of the Association Between Dietary Inflammation Index and C‐Reactive Protein Level.” Medicine 103, no. 19: e38196. 38728463 10.1097/MD.0000000000038196 PMC11081557

[fsn371501-bib-0020] Javed, A. A. , J. Ma , L. N. Anderson , et al. 2022. “Age‐Appropriate BMI Cut‐Points for Cardiometabolic Health Risk: A Cross‐Sectional Analysis of the Canadian Longitudinal Study on Aging.” International Journal of Obesity 46, no. 5: 1027–1035. 35094005 10.1038/s41366-022-01069-4

[fsn371501-bib-0021] Kourlaba, G. , and D. B. Panagiotakos . 2009. “Dietary Quality Indices and Human Health: A Review.” Maturitas 62, no. 1: 1–8. 19128905 10.1016/j.maturitas.2008.11.021

[fsn371501-bib-0022] Larson, R. B. 2019. “Controlling Social Desirability Bias.” International Journal of Market Research 61, no. 5: 534–547.

[fsn371501-bib-0023] Lécuyer, L. , N. Laouali , V. Viallon , et al. 2023. “Associations Between Dietary Inflammatory Scores and Biomarkers of Inflammation in the European Prospective Investigation Into Cancer and Nutrition (EPIC) Cohort.” Clinical Nutrition 42, no. 7: 1115–1125. 37271707 10.1016/j.clnu.2023.05.012

[fsn371501-bib-0024] Li, Y. , and L. Yuhong . 2024. “Higher Dietary Inflammatory Index Score Is Associated With a Greater Risk of High Allostatic Load in US Adults: National Health and Nutrition Examination Survey, 2017–2020.” Journal of the Academy of Nutrition and Dietetics 125: 909–921.e2. 39667434 10.1016/j.jand.2024.12.006

[fsn371501-bib-0025] Linton, C. , M. A. Schaumberg , and H. H. Wright . 2024. “Dietary Inflammatory Index Is Not Associated With Bone Mineral Density in Functionally Able Community‐Dwelling Older Adults.” European Journal of Nutrition 63: 1–11. 10.1007/s00394-024-03500-0 PMC11519128 39317870

[fsn371501-bib-0026] Linton, C. , H. H. Wright , D. P. Wadsworth , and M. A. Schaumberg . 2022. “Dietary Inflammatory Index and Associations With Sarcopenia Symptomology in Community‐Dwelling Older Adults.” Nutrients 14, no. 24: 5319. 36558478 10.3390/nu14245319 PMC9787040

[fsn371501-bib-0027] Lombard, M. J. , N. P. Steyn , K. E. Charlton , and M. Senekal . 2015. “Application and Interpretation of Multiple Statistical Tests to Evaluate Validity of Dietary Intake Assessment Methods.” Nutrition Journal 14: 1–11. 25897837 10.1186/s12937-015-0027-y PMC4471918

[fsn371501-bib-0028] Mao, Y. , J. Weng , Q. Xie , et al. 2024. “Association Between Dietary Inflammatory Index and Stroke in the US Population: Evidence From NHANES 1999–2018.” BMC Public Health 24, no. 1: 50. 38166986 10.1186/s12889-023-17556-w PMC10763382

[fsn371501-bib-0029] Mazidi, M. , N. Shivappa , M. Wirth , J. Hebert , H. Vatanparast , and A. Kengne . 2017. “The Association Between Dietary Inflammatory Properties and Bone Mineral Density and Risk of Fracture in US Adults.” European Journal of Clinical Nutrition 71, no. 11: 1273–1277. 29019343 10.1038/ejcn.2017.133

[fsn371501-bib-0030] McLennan, W. , and A. S. Podger . 1998. National Nutrition Survey: Nutrient Intakes and Physical Measurements, Australia, 1995. Australian Bureau of Statistics.

[fsn371501-bib-0031] Moshfegh, A. J. , D. G. Rhodes , D. J. Baer , et al. 2008. “The US Department of Agriculture Automated Multiple‐Pass Method Reduces Bias in the Collection of Energy Intakes.” American Journal of Clinical Nutrition 88, no. 2: 324–332. 18689367 10.1093/ajcn/88.2.324

[fsn371501-bib-0032] Naska, A. , A. Lagiou , and P. Lagiou . 2017. “Dietary Assessment Methods in Epidemiological Research: Current State of the Art and Future Prospects.” F1000Research 6: 926. 28690835 10.12688/f1000research.10703.1 PMC5482335

[fsn371501-bib-0033] Neale, E. P. , Y. C. Probst , and L. C. Tapsell . 2016. “Development of a Matching File of Australian Food Composition Databases (AUSNUT 2007 to 2011–13).” Journal of Food Composition and Analysis 50: 30–35.

[fsn371501-bib-0034] Norton, K. , and L. Norton . 2011. “Pre‐Exercise Screening. Guide to the Australian Adult Pre‐Exercise Screening System Exercise and Sports Science Australia.”

[fsn371501-bib-0035] Orchard, T. , V. Yildiz , S. E. Steck , et al. 2017. “Dietary Inflammatory Index, Bone Mineral Density, and Risk of Fracture in Postmenopausal Women: Results From the Women's Health Initiative.” Journal of Bone and Mineral Research 32, no. 5: 1136–1146. 28019686 10.1002/jbmr.3070 PMC5413384

[fsn371501-bib-0036] Poslusna, K. , J. Ruprich , J. H. de Vries , M. Jakubikova , and P. van't Veer . 2009. “Misreporting of Energy and Micronutrient Intake Estimated by Food Records and 24 Hour Recalls, Control and Adjustment Methods in Practice.” British Journal of Nutrition 101, no. S2: S73–S85. 19594967 10.1017/S0007114509990602

[fsn371501-bib-0037] Santos‐Báez, L. S. , M. N. Ravelli , D. A. Díaz‐Rizzolo , et al. 2025. “Dietary Misreporting: A Comparative Study of Recalls vs Energy Expenditure and Energy Intake by Doubly‐Labeled Water in Older Adults With Overweight or Obesity.” BMC Medical Research Methodology 25, no. 1: 115. 40287632 10.1186/s12874-025-02568-4 PMC12034172

[fsn371501-bib-0038] Shivappa, N. , J. R. Hébert , M. Karamati , S.‐E. Shariati‐Bafghi , and B. Rashidkhani . 2016. “Increased Inflammatory Potential of Diet Is Associated With Bone Mineral Density Among Postmenopausal Women in Iran.” European Journal of Nutrition 55, no. 2: 561–568. 25778389 10.1007/s00394-015-0875-4

[fsn371501-bib-0039] Shivappa, N. , S. E. Steck , T. G. Hurley , J. R. Hussey , and J. R. Hebert . 2014a. “Designing and Developing a Literature‐Derived, Population‐Based Dietary Inflammatory Index.” Public Health Nutrition 17, no. 8: 1689–1696. 10.1017/S1368980013002115. 23941862 PMC3925198

[fsn371501-bib-0040] Shivappa, N. , S. E. Steck , T. G. Hurley , et al. 2014b. “A Population‐Based Dietary Inflammatory Index Predicts Levels of C‐Reactive Protein in the Seasonal Variation of Blood Cholesterol Study (SEASONS).” Public Health Nutrition 17, no. 8: 1825–1833. 24107546 10.1017/S1368980013002565 PMC3983179

[fsn371501-bib-0041] Slimani, N. , H. Freisling , A. K. Illner , and I. Huybrechts . 2015. “Methods to Determine Dietary Intake.” In Nutrition Research Methodologies. John Wiley & Sons, Ltd. 48–70.

[fsn371501-bib-0042] Subar, A. F. , V. Kipnis , R. P. Troiano , et al. 2003. “Using Intake Biomarkers to Evaluate the Extent of Dietary Misreporting in a Large Sample of Adults: The OPEN Study.” American Journal of Epidemiology 158, no. 1: 1–13. 12835280 10.1093/aje/kwg092

[fsn371501-bib-0043] Tabung, F. K. , S. E. Steck , J. Zhang , et al. 2015. “Construct Validation of the Dietary Inflammatory Index Among Postmenopausal Women.” Annals of Epidemiology 25, no. 6: 398–405. 25900255 10.1016/j.annepidem.2015.03.009 PMC4433562

[fsn371501-bib-0044] Vahid, F. , N. Shivappa , Z. Faghfoori , et al. 2018. “Validation of a Dietary Inflammatory Index (DII) and Association With Risk of Gastric Cancer: A Case‐Control Study.” Asian Pacific Journal of Cancer Prevention 19, no. 6: 1471–1477. 29936717 10.22034/APJCP.2018.19.6.1471 PMC6103570

[fsn371501-bib-0045] Weisstein, E. W. 2004. “Bonferroni Correction.” https://mathworld.wolfram.com/.

[fsn371501-bib-0046] Wirth, M. D. , N. Shivappa , L. Davis , et al. 2017. “Construct Validation of the Dietary Inflammatory Index Among African Americans.” Journal of Nutrition, Health & Aging 21, no. 5: 487–491. 10.1007/s12603-016-0775-1 PMC5547883 28448077

[fsn371501-bib-0047] Xu, Z. 2025. “Improving the Dietaryindex R Package: A Proposal to Include Additional Components for More Accurate DII Computation in NHANES.” American Journal of Clinical Nutrition 121, no. 1: 174–175. 39528051 10.1016/j.ajcnut.2024.10.023

[fsn371501-bib-0048] Zhai, Y. , F. Hu , L. Yuan , et al. 2024. “Associations Between an Energy‐Adjusted Inflammatory Diet Index and Incident Depression: A Cohort Study.” British Journal of Nutrition: 1–10. 10.1017/S0007114524002253 39501636

[fsn371501-bib-0049] Zhao, C. , M. Lin , Y. Yang , et al. 2025. “Association Between Dietary Inflammatory Index and Cardiovascular–Kidney–Metabolic Syndrome Risk: A Cross‐Sectional Study.” Nutrition Journal 24, no. 1: 60. 40221720 10.1186/s12937-025-01127-3 PMC11992876

